# Transesophageal Echocardiographic Evaluation of Coronary Blood Flow and the Initial Flap Assisting in the Surgical Decision-Making: A Case of Acute Type A Aortic Dissection

**DOI:** 10.7759/cureus.61872

**Published:** 2024-06-07

**Authors:** Keisuke Sumii, Takuya Ichimura, Hideyuki Nakagawa, Akira Kitamura

**Affiliations:** 1 Anesthesiology, Saitama International Medical Center, Saitama Medical University, Saitama, JPN

**Keywords:** pulsed doppler, transesophageal echocardiography, coronary artery blood flow, aortic flap, aortic dissection

## Abstract

Acute aortic dissection is a life-threatening condition. Myocardial ischemia associated with dissection occurs due to direct extension into the coronary artery or indirect involvement of the coronary ostia secondary to the dissection flap. Thus, the surgical procedure may require coronary reconstruction, in addition to aortic replacement. We experienced a case in which coronary artery reconstruction could be avoided because intraoperative transesophageal echocardiography showed that the aortic flap did not obstruct the right coronary artery in systole, and pulsed Doppler imaging indicated that there was sufficient coronary blood flow. This case shows that it is critical to establish a correct and early diagnosis and to proceed with the appropriate treatment for patients with myocardial ischemia.

## Introduction

Acute type A aortic dissection (ATAAD) is a challenging medical emergency that is associated with increased mortality, even when recognized early and treated appropriately. ATAAD may be accompanied by aortic valve dysfunction; coronary, cerebral, and visceral malperfusion; and a markedly increased risk of aortic rupture [1,2]. In such cases, myocardial ischemia associated with dissection is critical and occurs due to direct extension of the coronary artery or indirect involvement of the coronary ostia secondary to the dissection flap. In this situation, the surgical procedure may require coronary reconstruction, in addition to aorta replacement. Criteria for the procedure for ATAAD extending to the coronary artery origin have been proposed by Neri et al. [3], but these criteria are not completely clear. Since transesophageal echocardiography (TEE) can sometimes provide an accurate diagnosis for coronary aortic dissection with a coronary false channel, we examined the coronary flow pattern in the systolic and diastolic phases. Here, we report a case in which intraoperative TEE findings allowed a decision on the surgical procedure.

## Case presentation

**Figure 1:**
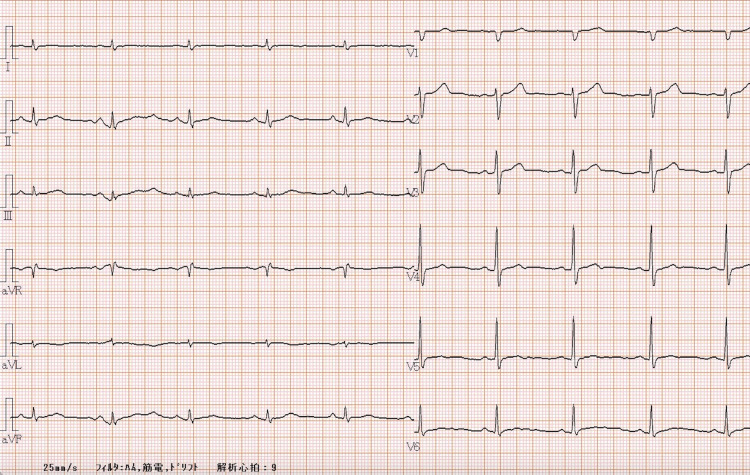
12-lead electrocardiogram (ECG) on admission. ECG depicted in this figure demonstrates a normal sinus rhythm with no ST-segment changes.

The patient gave consent to the publication of this case report. A 65-year-old man (171 cm, 68.5 kg) was diagnosed with ATAAD and underwent emergency surgery. When the patient was hospitalized, his consciousness was clear, blood pressure was 136/60 mmHg, and heart rate was 64 beats/min. A preoperative electrocardiogram showed sinus rhythm with no ST-segment changes (Figure 1). Preoperative transthoracic echocardiography (TTE) showed good cardiac contraction, no aortic valve regurgitation, no aortic flap, and no cardiac tamponade. Contrast-enhanced CT showed dissection from the aortic root to the common iliac artery. The superior iliac artery and right renal artery were perfused from the false lumen, but other arterial branches were perfused from the true lumen. After admission, emergency surgery was performed on the same day.

Anesthetic course

Anesthesia was induced with midazolam and fentanyl and maintained with remifentanil and sevoflurane. From intra- to postoperatively, circulation was maintained with dopamine at 1.5-2.9 µg/kg/min, norepinephrine at 0.03-0.06 µg/kg/min, and epinephrine at 0.01-0.04 µg/kg/min. 

Surgical course

**Figure 2:**
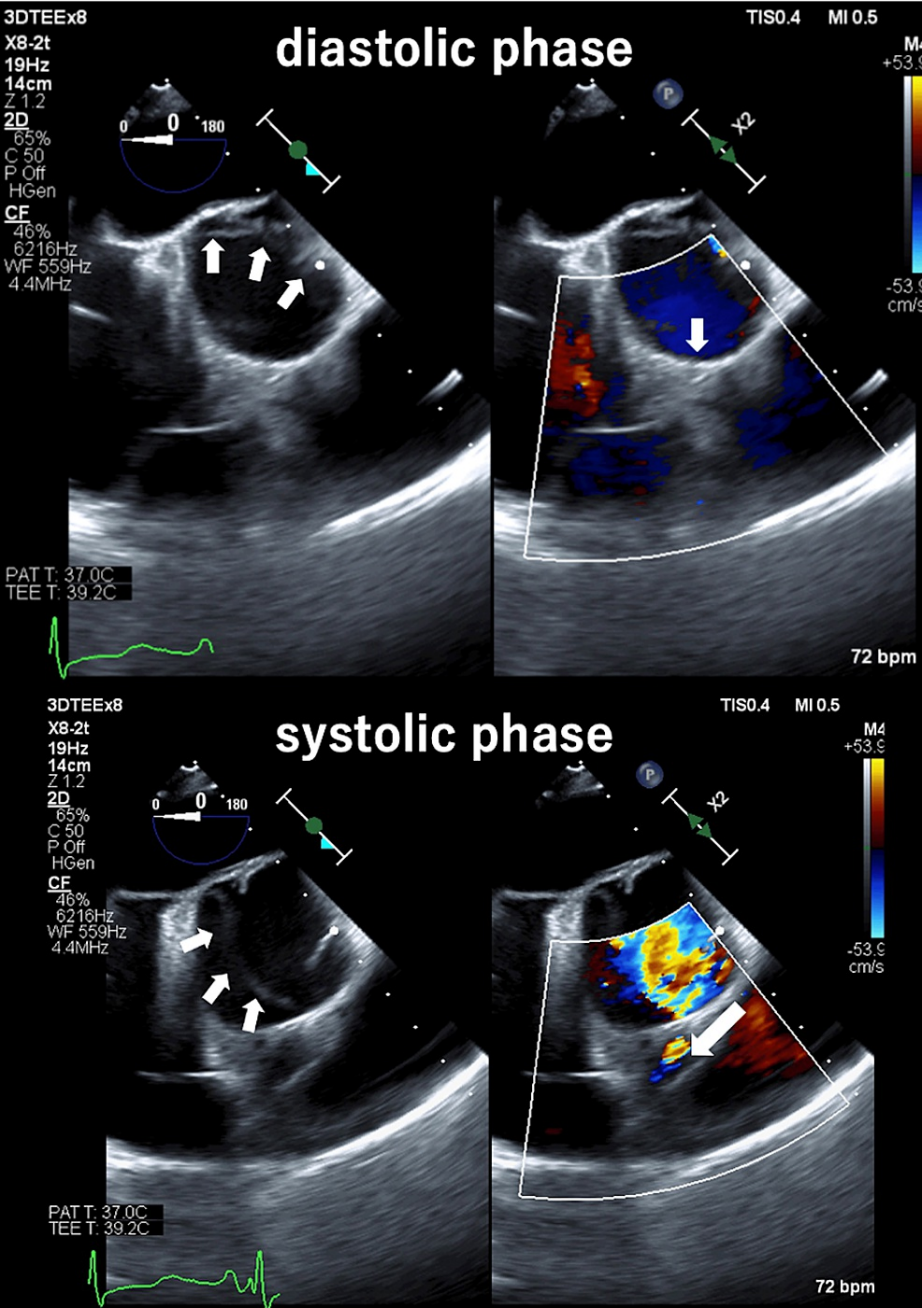
Transesophageal echocardiographic images after induction of anesthesia (aortic valve level, short axis view: left panel: B-mode, right panel: color Doppler). The right coronary artery origin was occluded during diastole by the aortic flap (upper row). Arrows in upper left panel: aortic flap; arrow in upper right panel, right coronary artery origin. The right coronary artery origin was occluded during the diastolic phase by the aortic flap, but this was released during the systolic phase (lower row). Arrows in lower left panel: aortic flap, arrow in lower right panel: direction of blood flow in the right coronary artery.

**Figure 3:**
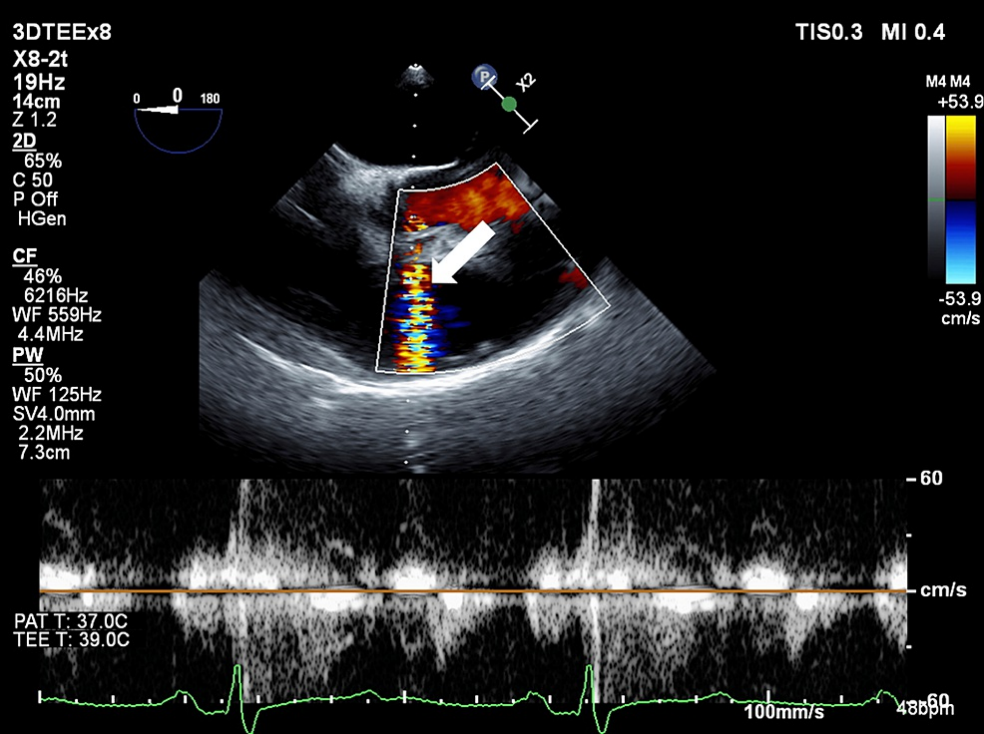
Evaluation of right coronary artery blood flow by pulsed Doppler in a transesophageal echocardiographic image (aortic valve level, short axis view) after induction of anesthesia (arrow: direction of blood flow in the right coronary artery).

After anesthesia induction, mild enlargement of the aortic base was observed on TEE, but there was no aortic valve regurgitation. The right coronary artery origin was occluded by the aortic flap during diastole, but the origin was released during systole (Figure 2). The left coronary artery origin was not occluded. Evaluation of right coronary artery blood flow by pulsed Doppler imaging showed no abnormal flow pattern during systole and diastole (Figure 3). There was also no abnormal wall motion or abnormal electrocardiogram pattern during surgery. After consultation with the surgeon about these negative findings, it was decided to perform aortic root, ascending, and arch replacement without coronary artery reconstruction.

Postoperative course

The patient was extubated on postoperative day (POD) two and left the ICU without any ECG abnormality for four postoperative days. Subsequently, contrast-enhanced CT confirmed false lumen closure and TTE showed no abnormal wall motion. The patient was discharged on POD 33 with no problems in respiratory or circulatory dynamics during hospitalization. Follow-up contrast CT conducted at one week and three, six, and 12 months after surgery suggested a thrombus-like false channel and no major change in the aortic diameter.

## Discussion

The patient had no findings of coronary malperfusion on preoperative examinations, and emergency surgery was performed with neither coronary angiography nor prior percutaneous coronary intervention (PCI). Intraoperative TEE showed that the right coronary artery was occluded by an aortic flap at its entrance during diastole, but the flap was released during systole. An accurate assessment of right coronary artery blood flow in the systolic and diastolic phases using pulsed Doppler imaging showed no findings of interrupted coronary artery blood flow or significant accelerated blood flow that would suggest stenosis. There was also no abnormal wall motion or electrocardiogram pattern. Therefore, we suggested to the surgeon that coronary artery bypass grafting (CABG) was probably not necessary as an additional procedure. Actually, the surgeon reported no visual findings of coronary artery obstruction due to a flap. 

**Figure 4:**
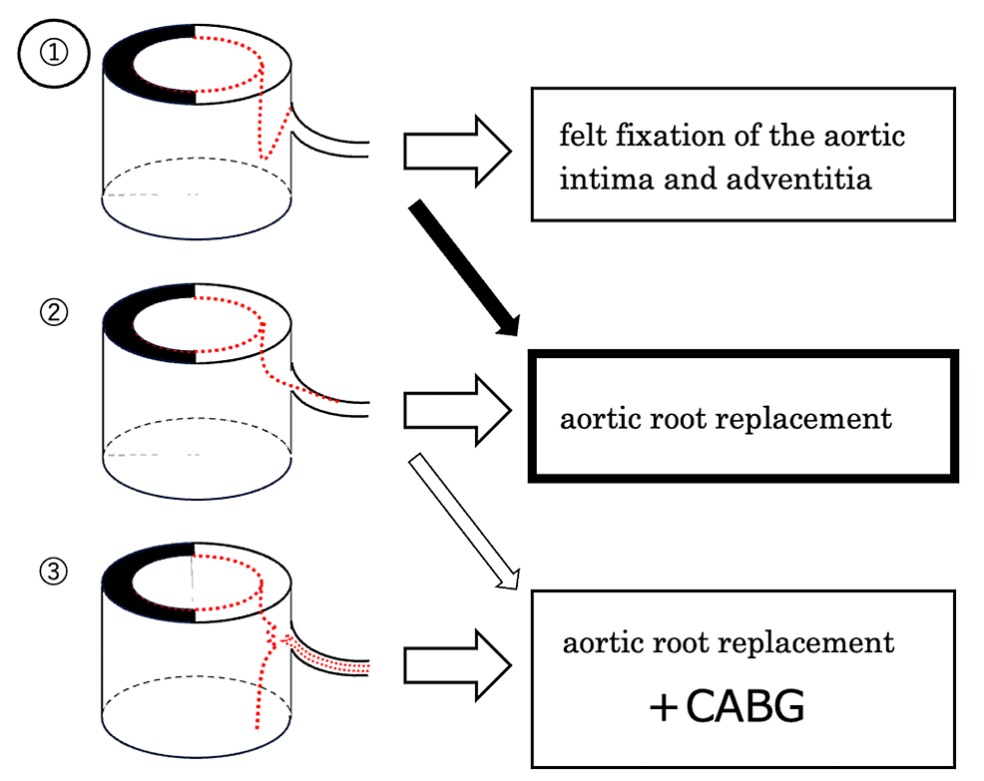
Kreibich et al. described methods for reconstruction of the aortic root for each type of ATAAD. ① The aortic flap obstructs the coronary artery entrance, but the coronary artery itself does not have a dissected lumen. ② The dissected lumen extends into the lumen of the coronary artery. ③ The coronary artery is completely separated from the aortic root. In this case, the aortic root was type ① and an aortic root replacement was performed (black arrow). Source: Reference [6]. ATAAD: acute type A aortic dissection, CABG: coronary artery bypass grafting.

Güllü et al. [4] reported that TEE was useful for diagnosis and treatment and allowed confirmation of intermittent occlusion of the left main coronary artery by an aortic flap associated with ATAAD; however, evaluation of coronary blood flow using pulsed Doppler imaging was not performed [4]. As in this case, aortic dissection may progress to where the dissection lumen extends around the coronary artery inlet both intra- and postoperatively [5]. Therefore, there is an urgent need to confirm by TEE that the dissection lumen does not extend into the coronary artery and that the coronary artery has not been completely torn, regarding Neri's classification [3]. A retrospective observational study showed that if the coronary artery does not have a dissected lumen, felt fixation of the aortic intima and adventitia can be performed, whereas if the coronary artery has a dissected lumen or is completely separated from the aortic root, the aortic root replacement and CABG are required (Figure 4) [6]. In addition to the need for CABG, the timing of CABG should also be considered. Preemptive CABG reduces the time of coronary perfusion insufficiency and provides reliable cardiac arrest with progressive administration of cardioprotective fluid during cardiopulmonary bypass. Stanger et al. [7] and Gillinov et al. [8] reported good results with aortic root replacement after adequate evaluation of coronary perfusion, including grafts, with preemptive CABG. On the other hand, a less experienced surgeon may perform CABG under beating heart difficulty, and it is necessary to discuss this issue with the team. While the technique of coronary anastomosis is relatively easy, it does prolong the duration of cardiopulmonary bypass and coagulation disorder.

In addition to TEE, electrocardiography and coronary angiography are useful for determining the need for CABG. TEE can detect coronary ischemia by evaluating ventricular wall motion and confirming obstruction of the coronary artery origin by the aortic flap [9]. If the coronary artery origin is occluded by the aortic flap, the blood flow pattern in the right coronary artery must be confirmed over both the systolic and diastolic phases [10,11]. Coronary artery blood flow can be evaluated quantitatively in real time by pulsed Doppler imaging. Acceleration of blood flow at a stenotic site is significantly faster than that at non-stenotic sites in the coronary arteries; thus, the blood flow velocity has been measured as 143 cm s^−1^ at a site with >50% stenosis, compared to 38 cm s^−1^ at a non-stenotic site [12]. Diastolic maximum and minimum velocities in the coronary artery may also be lower in patients with impaired perfusion compared to those with normal perfusion [13], although other findings have indicated no changes in these velocities in the presence of perfusion impairment [14]. This makes it difficult to determine the presence of perfusion impairment using velocities in the coronary artery itself. 

The "Guidelines for the Treatment of Aortic Aneurysm and Dissection 2020" issued by the Japanese Circulation Society state that "the presence of myocardial ischemic changes should be evaluated by 12-lead electrocardiography before surgery," in addition to evaluation of the presence of abnormal wall motion by echocardiography. In these guidelines, it is recommended in Class I cases that myocardial ischemia should be evaluated using intraoperative transesophageal echocardiography to make a diagnosis of coronary artery malperfusion in patients with acute aorta dissection [15]. However, pre- and intraoperative abnormal electrocardiography may show non-specific coronary ischemia-like changes, such as coronary artery spasm and decreased coronary artery blood flow associated with hypotension. Therefore, care is required to avoid evaluation of these changes in isolation [16]. Preoperative coronary angiography should not be performed because of the risk of aortic rupture due to the procedure and possible delay in surgical intervention, although it is likely to provide accurate information [17]. Recently, an early coronary reperfusion strategy for ATAAD with coronary malperfusion has been proposed [18,19], but the best approach remains controversial. 

It is difficult to evaluate the coronary artery origin in a patient with an aortic root with calcification or an artificial valve. Measurement of coronary blood flow velocity by pulsed Doppler is angle-dependent, which makes it particularly difficult to evaluate the left coronary artery. If the angle between the ultrasound beam and blood flow is defined as θ, angle correction is recommended when θ is >30°. A 3.2-degree shift in the angle correction produces a 5% error. For θ >60°, angle correction does not provide sufficient accuracy for measuring coronary blood flow velocity [20]. In addition, there are no clear standards for the procedure and timing of coronary artery reconstruction with aortic root replacement in ATAAD, and further study is needed. Evaluation of the advantages and disadvantages of each procedure is required, and it is important to decide after careful consultation with the surgeon when PCI or CABG and aortic root replacement are performed simultaneously.

## Conclusions

We experienced a case in which coronary artery reconstruction could be avoided because intraoperative transesophageal echocardiography showed that the aortic flap did not obstruct the right coronary artery in systole, and the pulsed Doppler imaging indicated that there was sufficient coronary blood flow.

In the case of ATAAD, we confirmed the release of an aortic flap occlusion at the origin of the right coronary artery and evaluated coronary artery blood flow using pulsed Doppler imaging. This was helpful in determining the most appropriate treatment.
